# Adult adipose-derived stem cells and breast cancer: a controversial relationship

**DOI:** 10.1186/2193-1801-3-345

**Published:** 2014-07-08

**Authors:** Alessandra Bielli, Maria Giovanna Scioli, Pietro Gentile, Sara Agostinelli, Chiara Tarquini, Valerio Cervelli, Augusto Orlandi

**Affiliations:** Anatomic Pathology, Tor Vergata University of Rome, Via Montpellier, 00133 Rome, Italy; Plastic Surgery, Department of Biomedicine and Prevention, Tor Vergata University, Rome, Italy

**Keywords:** Adipose-derived stem cell, Stromal vascular fraction, Fat grafting, Breast cancer, Carcinogenesis, Breast reconstruction, Akt

## Abstract

Breast cancer is the most common cancer in women and autologous fat grafting is an important clinical application in treatment of post-surgical deformities. The simplicity of fat grafting procedures and the absence of subsequent visible scar prompted an increasing interest for this technique. The plasticity of adipose-derived stem cells (ASCs) obtained from stromal vascular fraction (SVF) of adult adipose tissue provided exciting perspectives for regenerative medicine and surgery. The recent discovery that SVF/ASC enrichment further ameliorates clinical efficacy of grafting ASCs suggest as ASC-mediated new adipogenesis and vasculogenesis. ASC adipogenic differentiation involves Akt activity and EGFRs, FGFRs, ERbB2 receptor-mediated pathways that also play a pivotal role in the regulation of breast cancer growth. Moreover, the finding that platelet-derived growth factors and hormones improved long-term maintenance of fat grafting raises new concerns for their use during breast reconstruction after cancer surgery. However, it remains unclear whether grafted or resident ASCs may increase the risk of *de novo* cancer development or recurrence. Preliminary follow-up studies seem to support the efficacy and safety of SVF/ASCs enrichment and the additional benefit from the combined use of autologous platelet-derived growth factors and hormones during breast reconstruction procedures. In the present review we highlighted the complex interplay between resident or grafted ASCs, mature adipocytes, dormant or active breast cancer cells and tumor microenvironment. Actually, data concerning the permissive role of ASCs on breast cancer progression are contrasting, although no clear evidence speaking against their use exists.

## Introduction

**Figure 1:**
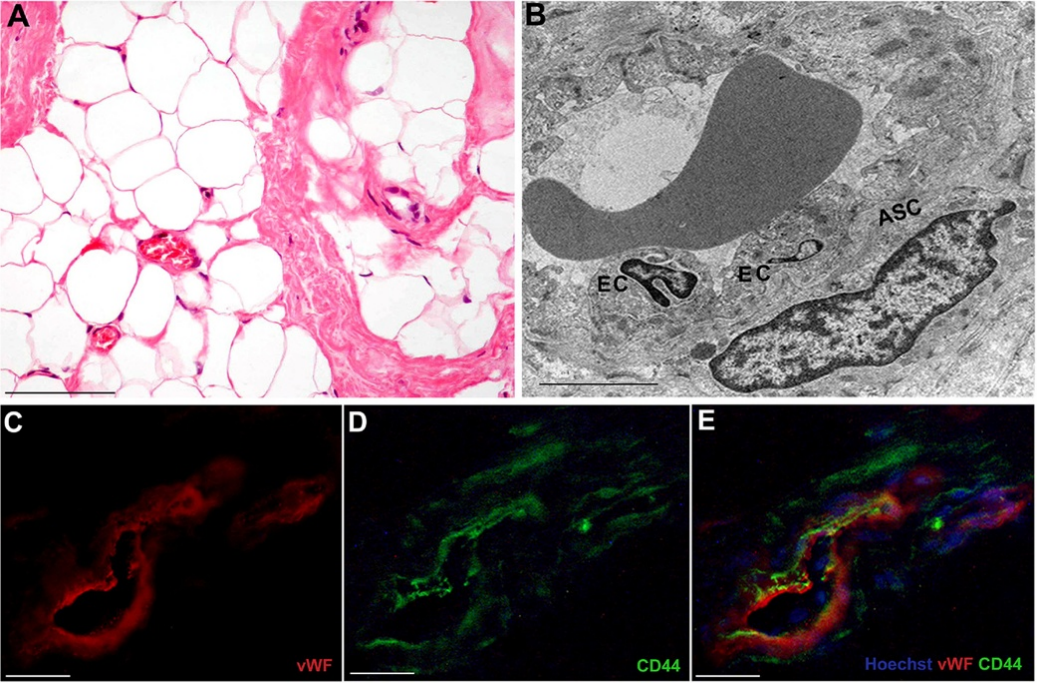
**Microscopic characterization of human breast adipose tissue. A**, Normal mammary adipose tissue after Haematoxylin and Eosin staining. Scale bar, 100 μm. **B**, transmission electron microscopy image of human breast adipose tissue showing perivascular adipose-derived stem cells (ASC) surrounding a small blood vessel with endothelial cells (EC). Scale bar, 10 μm. **C**, Under fluorescence microscopy, vascular endothelial cells are vonWillenbrand positive (red fluorescence) in the breast adipose tissue, whereas **D**, adipose-derived stem cell are CD44 positive (green fluorescence). **E**, Merged image shows that CD44 positive cells reside around endothelial cells of small blood vessels in breast adipose tissue. Nuclei are stained with Hoechst. Scale bar, 100 μm.

Adult adipose tissue is a multifunctional organ that contains various cellular types, including mature adipocytes, macrophages and stromal cells, supported by connective tissue surrounding fine capillaries (Zuk et al. 2001; Gentile et al. 2012a; Tran et al. 2012). When isolated *in vitro*, stromal cells have the potential to form bone, cartilage, muscle and fat tissues and have been variously termed, including preadipocytes and multipotent adipose-derived stem cells. However, the term “adipose-derived stem cells” (ASCs) has been successively recommended for the consistency between research groups (Zhao et al. 2010). In adult adipose tissue (Figure 1A), ASCs are considered to reside between mature adipocytes and extracellular matrix around small vessels (Tran et al. 2012). In fact, transmission electron microscopy of human breast adipose tissue showed the presence of cells featuring ASCs arranged around the endothelial cells of small vessels (Figure 1B), strongly supporting their perivascular origin (Traktuev et al. 2008; Crisan et al. 2008). ASCs likely contribute to adipose tissue cell turn-over (Strawford et al. 2004; Wang et al. 2012a). ASCs can be isolated from subcutaneous adult adipose tissue after liposuction by enzymatic digestion (Gimble et al. 2007; Cervelli et al. 2009). After centrifugation, the obtained heterogeneous mixture of endothelial cells, smooth muscle cells, fibroblast, pericytes, mast cells and preadipocytes is named stromal-vascular fraction (SVF). ASCs can be separated from SVF by adhesion on plastic dishes (Gimble et al. 2007; Cervelli et al. 2009). Before discovering of the plasticity of ASCs, bone marrow was clinically considered the major tissue source of human adult stem cells, the so-called mesenchymal stem cells (MSCs) (Izadpanah et al. 2006). ASCs and MSCs share the ability to differentiate along multiple lineage pathways, including vasculogenetic properties. Cells with stem phenotype and vasculogenetic capacities have been also identified in the circulatory system, in the vessel wall and in various extravascular sites (Grenier et al. 2007; Orlandi & Bennett 2010). Furthermore, ASCs can easily differentiate in mature adipocytes, and adipogenic differentiation is greatly increased by combined treatment with insulin and platelet-derived growth factors, with an increased long-term maintenance of fat grafts (Cervelli et al. 2012). Autologous fat grafting with SVF enrichment for regenerative surgical purposes, in particular in the therapy of post-traumatic lower extremity ulcers, give promising results (Cervelli et al. 2011). Similarly, SVF enrichment allows fat graft maintenance, likely favoring vascularization and collagen synthesis activity (Gentile et al. 2012b). These findings suggest the innovative use of the SVF/ASC enrichment and growth factors also in the breast reconstruction to avoid the frequent complications of fat grafting, including fat necrosis, cyst formation and calcification (Gutowski 2009). Adipose tissue is extremely metabolically active, as documented by its capacity to secrete hormones, growth factors and cytokines by both mature adipocytes and ASCs (Fruhbeck et al. 2001; Kilroy et al. 2007). Similarly, the discovery of ASCs as main actors in the regulatory scenario of adipose tissue cell turn-over requires further attention for the potential interplay between resident, grafted ASCs and residual breast cancer cells or adjacent *in situ* lesions. This finding induced caution and suggested some concerns about the use of fat grafting with SVF/ASC enrichment in breast reconstruction following cancer surgery. In the present review, we tried to describe the biomolecular pathways regulating proliferation and differentiation of ASCs, in order to define potential implications of breast cancer cell biology and risks for their use in post-surgery breast cancer reconstruction.

## Phenotypic characterization of adipose-derived stem cells

**Figure 2:**
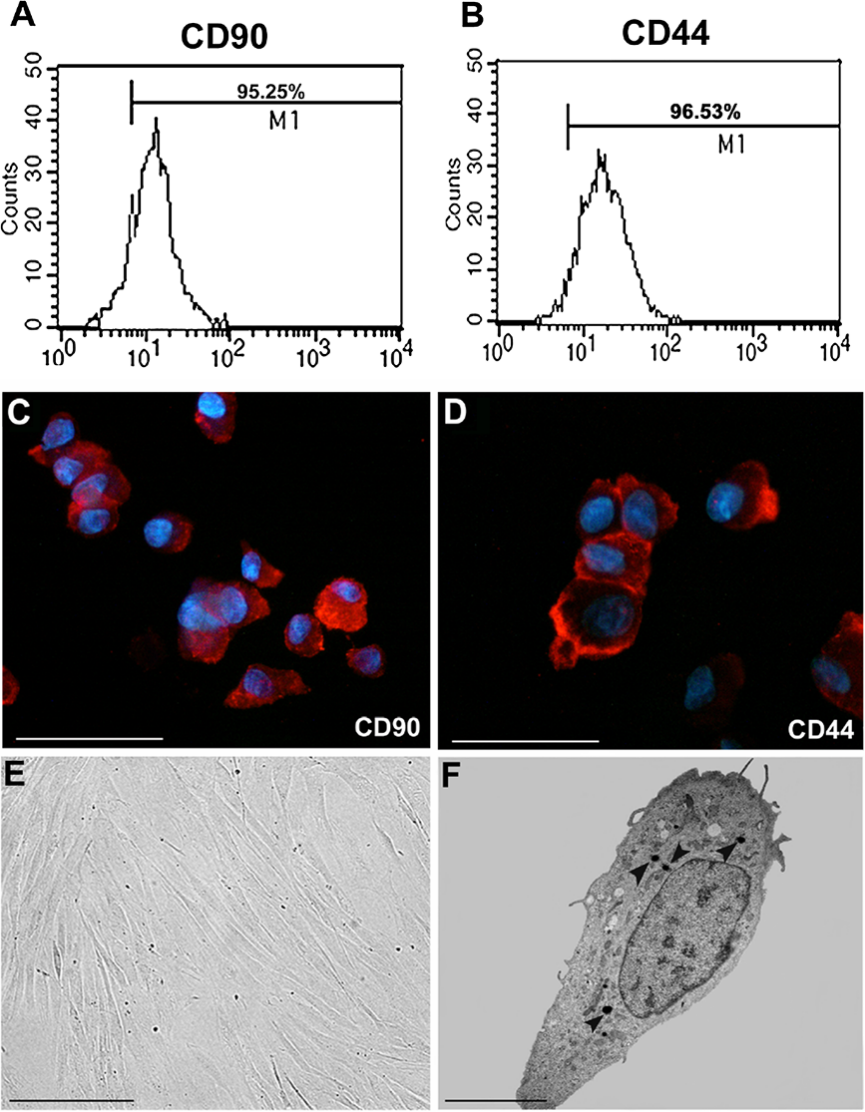
**Phenotypic analysis of human adipose-derived stem cells. A** and **B**, Flow cytometry depicting the diffuse expression of CD90 and CD44 stromal markers. **C** and **D**, Immunofluorescence staining revealing the strong expression of CD44 and CD90 in cultured ASCs. Nuclei are stained with Hoechst. Scale bar, 50 μm **E**, Phase contrast micrograph shows the typical elongated shape of adipose-derived stem cells in serum cultures. Scale bar, 150 μm **F**, Transmission electron microscopy image showing adipose-derived stem cell with the presence of intracytoplasmic lipid droplet electron dense (arrow heads). Scale bar, 10 μm.

ASCs share with MSCs the differentiation potential along several mesenchymal lineages (Gimble et al. 2007) (Peng et al. 2008). Nevertheless, some characteristics of ASCs, in particular the maintenance of proliferating ability in culture, are even greater than those of MSCs (Xu et al. 2005). The surface antigen profile of ASCs isolated from human adipose tissue, changes *in vitro* as a function of time and/or passage in culture (Mitchell et al. 2006). Table 1 summarizes the antigenic profile of ASCs. After two or more passages *in vitro*, ASC surface immunophenotype resembles that of MSCs, with a similarity greater than 90% (Gimble et al. 2007). Nevertheless, some differences in surface protein expression have been described. The presence of the glycoprotein CD34 on the surface of human ASCs is not reported in MSCs (Pittenger et al. 1999). Since CD34 is abundantly expressed in human ASCs, immunofluorescence makes possible to identify ASCs as CD34^+^ cells and to differentiate them from circulating precursors (Pittenger et al. 1999), confirming in human adipose tissue the presence of CD34^+^ cells and the perivascular origin of ASCs (Figure 1C-E). Cytofluorimetry and immunofluorescence represent suitable methods to investigate ASCs phenotype *in vitro* (Figure 2). Besides mesenchymal markers, such as CD44 and CD90, ASCs display pericytic markers, such as CD140a, CD140b and smooth muscle markers, such as α − smooth muscle actin (Traktuev et al. 2008).

**Table 1:** **Antigen profile of adipose-derived stem cell**

Antigen Category	Surface-positive Antigens
Cytoplasmic receptor	CD44 (hyaluronate), CD71 (transferrin)
Cell adhesion molecules	CD9, CD29, CD49 days, CD54, CD105,CD166
Extracellular matrix markers molecule	CD90, CD146, collagen types I and II, osteopontin, osteonectin
Stromal markers	CD29, CD44, CD73, CD90, CD166
Cytoskeleton markers	α-smooth muscle actin, vimentin, calponin*, caldesmin*
Stem cell markers	CD34, ABCG2

*After 7-days TGF-β1 treatment.

## Adipose-derived stem cells and angiogenesis

The fascinating differentiative pluripotency of ASCs and their ability to enhance vascularization (Bertolini et al. 2012; Merfeld-Clauss et al. 2010) progressively increased interest for their use in tissue engineering and regenerative medicine. The perivascular origin of ASCs and the expression of pericytic markers first suggested a role in vascular homeostasis of adipose tissue (Maumus et al. 2011). When transplanted, ASCs have the capacity to maintain the viability of fat transplanted through the secretion of growth factors that improve tissue survival (Kolle et al. 2013). Recent studies indicated that ASCs like MSCs are capable to promote angiogenesis through secretion of growth factors, in particular VEGF (Kinnaird et al. 2004; Salgado et al. 2010). Angiogenesis is a crucial event for cancer growth, and VEGF secretion plays a pivotal role in this process (Tarallo et al. 2010). Stem cells contribute to vascular remodelling by synthesizing collagen and secreting vascular growth factors (Orlandi and Bennett 2010). So, the expression of VEGF receptors in ASCs should be taken into account for future additional new anti-angiogenic strategies (Cassinelli et al. 2012) in breast cancer. It is worth of noting that ASCs share with resident vascular stem cells the paracrine production of VEGF (Cervelli et al. 2012; Ferlosio et al. 2012) and the expression of VEGF receptors (Kinnaird et al. 2004; Salgado et al. 2010). Furthermore, ASCs secrete hepatocyte growth factor, tumor necrosis factor-α and nerve growth factor (Salgado et al. 2010). Nerve growth factor and precursors are capable of inducing vascular remodeling by modulating vascular cells apoptotic sensitivity (Campagnolo et al. 2014).

## Cross-talk between mature adipocytes and breast cancer cells

Many studies focused the relationship between mature adipocytes and breast cancer cells. Rat mature adipocytes affect the biological behavior of epithelial cells through the production of leptin, adiponectin, tumor necrosis factor-*α*, heparin-binding epidermal growth factor, insulin-like growth factor-II and adipsin (Manabe et al. 2003). Furthermore, mature adipocytes metabolize androgen to estrogen trough their own aromatase activity (Miller et al. 1998). Estrogen synthesis affects breast carcinoma cell growth by a paracrine mechanism (Miller et al. 1998). Since breast adipose tissue is the major site of estrogen biosynthesis, its local delivering is likely involved in cancer progression. As matter of fact, estrogen level in breast tissues results 10 times greater than in blood as a consequence of high aromatase activity and tumor cells release stimulatory factors that amplify aromatase expression (Sasaki et al. 2010). Breast tumor cells also influence and modify the surrounding tissue microenvironment. Recent *in vivo* and *in vitro* studies demonstrated relevant phenotypic changes in adipocytes surrounding breast cancer (Dirat et al. 2011). Murine and human mature adipocytes co-cultured with tumor cells exhibited changes of the number and size of lipid droplets, a decrease of adipose markers level and over-expression of IL6, leading to a more aggressive tumor behavior (Walter et al. 2009). Also, mature adipocytes adjacent to the tumor showed a reduction of the expression of PPARγ and lipid droplet accumulation (Chandler et al. 2012). Adipose tissue may also stimulate the growth and survival of breast tumor cells trough the secretion of adipokines, such as leptin and adiponectin (Bertolini et al. 2012). It appears evident that adipocytes surrounding breast cancer are subjected to significant transcriptional changes with a marked increased expression of endocrine-related factors, influencing the growth and survival of breast cancer cells, in a paracrine loop (Ghosh et al. 2010). These findings support the hypothesis of an intimate cross-talk between breast cancer cells and immediately adjacent adipose tissue cells.

## Adipose-derived stem cells and breast cancer

Differently from mature adipocytes, the interplay between resident mesenchymal cells, including ASCs, and breast epithelial cells is still partially unknown. In this respect, it is still unclear whether preadipocytes act differently from mature adipocytes. ASCs are located in perivascular niches and contribute to cell turn-over, vascular network for the maintenance of adipose tissue tropism (Strawford et al. 2004; Wang et al. 2012a) and to regulate stem cell homeostasis. Dynamic and reciprocal communication between epithelial and stromal compartments occurs during breast cancer progression, with the production and release of a large panel of cytokines, chemokines and growth factors which are essentials for the generation of a more favorable microenvironment for tumor growth (Dirat et al. 2011; Wiseman and Werb 2002). These signals are capable to induce the recruitment of several cells types including MSCs, so promoting cancer growth, metastasis and tumour stroma formation (Kucerova et al. 2013; Karnoub et al. 2007). Tumor microenvironment is heterogeneous and, in recent studies, the presence within tumor bulk of a cancer stem cell has been hypothesised. Cancer stem cells are defined as a subpopulation that constitute a small percentage of the tumor bulk and displayed analogies to normal stem cells, with both embryonic and adult aspects, supported by phenotypic (surface marker) and functional (metabolic enzymes and transporters) features and clonogenic potential (Donnenberg et al. 2013). In the primary tumor, cancer stem cell may arise from the transformation of resident stem cells or from dedifferentiation of differentiated tumor cells in response to specific microenvironmental signals (Park et al. 2014). The acquisition of cancer stem cell features may be a partial reminiscence of an embryonic phenotype with an increase of susceptibility to epithelial-mesenchymal transition, supporting greater tumor growth and invasiveness (Park et al. 2014). Cells shift from a epithelial-like to a spindle-like morphology, accompanied by the expression of CD44 and CD90 stem markers and the maintenance of an adult stem cell phenotype (Donnenberg et al. 2013; Park et al. 2014). Furthermore, epithelial breast cancer cells undergoing to epithelial-mesenchymal-transition showed mesenchymal features with loss of polarity and stem like spindle shape, that favor motility, invasiveness and survival (Hass & Otte 2012). In this context, ASCs may interact with breast cancer cells through the formation of gap junctions that allows intercellular communication and the exchange of low molecular weight compounds (Donahue et al. 2003). The presence of gap junctions correlate with a more malignant phenotype and greater tumor progression and they can modulate the metastatic potential of the breast cancer cells (Mandel et al. 2013). Thus, the inhibition of gap junctions could partially block the stem cell-mediated growth induction and CD90 expression in breast cells. Conflicting data are reported concerning the role of ASCs in cancer progression. As reported, ASCs express surface markers, such as CD44, able to anchor some matrix-metalloproteinases to the cell surface. This CD44-matrix-metalloproteinases association mediates the reorganization of extracellular matrix components (Hass and Otte 2012). Moreover, experiments *in vivo* and *in vitro* reported that ASCs favor tumor growth, increasing extracellular matrix deposition and vascularization, suggesting that ASCs may directly contribute to the dense network of fibroblasts and desmoplastic reaction surrounding breast cancer (Wang et al. 2012a). The desmoplastic reaction represents the stromal response to cancer cell infiltration and it is due to the disruption of the basement membrane and the inflammatory remodeling of the extracellular matrix (Pinilla et al. 2009). Desmoplastic reaction involves increased activity of tissue metalloproteinases and studies *in vitro* documented that co-culture of human ASCs and breast cancer cells induce high levels of metalloproteinases (Pinilla et al. 2009). Desmoplastic reaction also promotes myofibroblasts recruitment (De Wever et al. 2008; Karagiannis et al. 2012) and a large number of myofibroblasts are documented in the stromal compartment of invasive human breast cancers (Gehmert et al. 2010; Orimo et al. 2005). Myofibroblasts are stromal fibroblasts with both myocyte and fibroblast features (Orlandi et al. 1994). ASCs isolated from tumors also express high levels of alpha-smooth muscle actin, a well-known myofibroblastic marker (Tomasek et al. 2002). This suggests that ASCs could act as a potential source of tumor myofibroblasts.

To better clarify their role in cancer progression, studies *in vivo* and *in vitro* have been performed to verify ASCs influence on dormant tumor cells and on their growth and invasiveness. In literature is not yet clear whether dormant tumor cells are out of cell cycle, or persist in a dynamic state of proliferation and death. The transition between dormant and active states requires the presence of various signals, such as cytokines, hormones and growth factors (Donnenberg et al. 2010). Xenograft model experiments documented that grafted ASCs act in a different manner on active and dormant breast cancer cells. In fact, whereas the active cancer cells require growth factors and a new vascular network for the survival and invasiveness, the dormant cancer cells do not immediately require support of these factors. The latter are more autonomous and their growth is slower (Donnenberg et al. 2010; Zimmerlin et al. 2011). Consequently, these results indicate that grafted ASCs favour the growth of active, but not dormant, breast cancer cells. Moreover, ASCs transplantation or co-injected into mouse breast cancer model did not promote tumor growth or metastasis, and this inhibitory effect has been identified as the cause for ASC-dependent Poly ADP ribose polymerase cleavage, so inducing tumor cell apoptosis (Sun et al. 2009).

Altogether, these studies highlight the concept that resident ASCs and cancer cells may interact in a complex and dynamic fashion influencing the tumor behavior. Further studies are needed to better clarify this *in vivo* interaction and define how selectively stimulate ASCs regenerative function without influencing tumorigenesis.

## Similarities of growth and differentiative pathways of adipose-derived stem cells and breast cancer cells

The proliferative arrest and/or cell loss are potential limitations in regenerative surgery strategies. So, exogenous growth factors should provide the necessary microenvironmental signals to accelerate cell proliferation, extracellular matrix synthesis and tissue deposition (Chen et al. 2010). Various receptor pathways regulated ASC proliferation and differentiation. Epidermal growth factor receptors (EGFRs), fibroblast growth factor receptors (FGFRs) and ErbB tyrosine kinase receptor (ErbB) families are involved in growth control and differentiation of cancer stem cells (Flageng et al. 2013; Nguyen et al. 2013; Reed et al. 2012; Liu et al. 2009). Recent studies documented the presence of EGFR and ErbB2 transcripts and proteins in ASCs (Cervelli et al. 2012). Platelet-derived growth factors stimulated ASCs proliferation and improved the maintenance of breast fat grafting in patients affected by soft tissue defects (Cervelli et al. 2012), but did not affect adipogenic differentiation of ASCs *in vitro* (Cervelli et al. 2009). This suggests that the pathways regulating proliferation and differentiation of ASCs are partially distinct. Platelet-derived growth factors clinically ameliorated efficacy of fat grafting, likely favoring ASC proliferation (Cervelli et al. 2009). Insulin further increase long-term fat graft maintenance and greatly promoted adipogenic differentiation by increasing Akt activity and down-regulating the expression and activity of EGFR and ErbB2 receptors, without significant proliferative arrest of ASCs (Cervelli et al. 2012). Adipogenic differentiation also associated to the increased FGFR-2 and FGFR-1 transcript levels, suggests a complex receptor-mediated control of adipogenesis in ASCs (Cervelli et al. 2012). Although the clinical use of growth factors may improve long-term fat graft volume maintenance, growth factors may also influence the activity of resting cancer cells (Liu et al. 2009). As reported above, estrogen sustains growth in breast cancer through the transcriptional up-regulation of various growth factors, such as EGFR, and Akt phosphorylation (Liu et al. 2009). Moreover, a cross-talk between erbB and estrogen receptor-mediated signaling has been reported in tumor progression and resistance to endocrine therapy of breast cancer cells (Normanno et al. 2005). In addition, aberrant expression and activation of FGFR activity is involved in the progression of breast cancer (Grose et al. 2007). In particular, FGFR1 expression is associated with an early relapse and poor survival of breast cancer patients (Turner et al. 2010). *In vitro* data alone seem to suggest the caution in the local use of growth factors in addition to fat graft and further investigation of the interplay between ASCs and breast cancer cells should be performed also *in vivo.*

## Breast lipografting: clinical studies and follow-up

Autologous fat grafting is a procedure widely used in breast reconstruction after cancer surgical treatment (Gentile et al. 2012b; Coleman and Saboeiro 2007). Clinical trials documented the absence of significant differences between patients undergoing lipofilling after mastectomy compared to untreated group. To minimize adverse effects, many attempts have been made to improve long term fat graft maintenance. A recent series of cases of breast reconstruction performed using autologous fat graft documented that, after a 10 years follow-up, there is no increased risk of relapse or new cancer development (Delay et al. 2009). In another study, 321 patients after breast cancer surgery treatment were subjected to lipofilling treatment (Petit et al. 2012). After six month, patients with lipofilling showed no relapse compared to untreated group. Nevertheless, when the study focused on patients previously diagnosed with breast intraepithelial neoplasia, the lipofilling group displayed a slightly higher risk of local recurrence, although not statistically significant, compared to the untreated group (Petit et al. 2012). However, a similar study carried out on 158 patients subjected to fat grafting procedures after a history of breast cancer surgery, did not show any increase risk of relapse after 18 month of follow-up (Rietjens et al. 2011). Other works compare the local recurrence before and after lipotransfer in patients undergoing mastectomy, with no statically significant differences between groups (Rigotti et al. 2010). Preliminary data from a relatively small number of patients describe that SVF enrichment improves fat graft survival, with no evidence of breast malignant transformation (Kolle et al. 2013). A recently introduced new technique combine the use of autologous SVF enrichment with platelet-derived growth factors to improve fat grafting maintenance after breast reconstruction. A series of 23 patients with breast cancer undergoing post-surgical breast reconstruction with fat grafting-SVF and platelet-derived growth factors did not show increased risk of new cancer development after 1 year follow-up compared with the control group, but evidenced the ameliorated maintenance of breast volume (Cervelli et al. 2012). Although preliminary, these results seem to support that the addition of platelet-derived growth factors to lipografting induces a safe improvement of breast volume maintenance (Gentile et al. 2013), likely for the ability of platelet-derived growth factors to stimulate vascularization (Coleman and Saboeiro 2007; Gentile et al. 2013). Clinical studies with more patients and a longer follow-up are needed to confirm the safety of SVF/ASCs enrichment during fat grafting procedures with or without platelet-derived growth factors and hormones in breast cancer patients.

## New stem cell therapies and surgical breast cancer reconstruction

Conventional cancer therapies include surgery, chemotherapy and radiotherapy. A certain number of preclinical studies recently proposed the use of MSCs as candidates to deliver anti-cancer drugs. Chemokines secreted by breast tumor cells are capable of stimulating MSCs migration and recruitment, suggesting a potential role for MSCs as delivery agents for chemotherapeutic purposes in breast tumours *in vivo*. MSCs can be readily transduced via adenoviral, retroviral or lentiviral vectors without compromising the capability for differentiation or the expression of surface markers. Consequently, MSCs are potentially suitable for a gene approach in cancer therapy through the induction of a more chronic and slow release of drugs that are often limited by their toxicity or short life (Kucerova et al. 2008). Interferon-β is a powerful inhibitor of tumor cell growth, but to be efficacy it needs a dose higher than the maximally tolerated. *In vitro*, MSCs transduced with adenoviral vector carrying human Interferon-β and co-cultured with breast cancer cells induced the reduction of cancer cells growth (Studeny et al. 2004). The same effect occurred, *in vivo*, when Interferon-β − transfected MSC cells are injected intravenously in a xenograft breast cancer mouse model (Studeny et al. 2004). The most of gene approaches use cytosine deaminase/5-fluorocytosine and thymidine kinase/ganciclovir. Ganciclovir is an inhibitor of DNA polymerase and, after DNA incorporation, inhibits chain elongation (Matuskova et al. 2012). The combined use of yeast cytosine deaminase gene with 5-fluorocytosine allows the activation of 5-fluorouracile, a drug normally used in conventional chemotherapy (Matuskova et al. 2012). However, when ASCs are transduced with thymidine kinase/ganciclovir the growth of breast cancer cells was inhibited, but the latters have proved resistant to cytosine deaminase/5-fluorocytosine treatment. These opposite effects are linked to the capability of adult stem cells and tumor cells to communicate via gap junctions, determining both chemosensitivity than chemoresistance (Matuskova et al. 2012; Kucerova et al. 2012). In other works ASCs has been tested as vehicle to deliver tumor necrosis factor-α and to induce TRAIL-mediated apoptosis of cancer cells (Grisendi et al. 2010). TRAIL is a member of the tumor necrosis factor super-family that induces a selective apoptosis through the activation of death receptors in cancer target cells, with no relevant effects on healthy cells (Grisendi et al. 2010). More recently, PPARγ ligands were shown capable of stimulating the differentiation of several cancer cells types, including breast cancer cells lines, suggesting a therapeutic utility in breast cancer treatment. PPARγ ligands inhibit the expression of aromatase and hence estrogen biosynthesis in adipose tissue surrounding human breast cancer (Rubin et al. 2000). At present, most hormonal therapies for breast cancer aim to the inhibit estrogen receptor and/or aromatase activity of cancer cells (den Hollander et al. 2013). Tamoxifen is an antagonist of estrogen receptor widely used in therapy of breast cancer, but after several years of treatment clonal cell line tumors become unresponsive to the drug (Higgins and Stearns 2009). The mechanism that underly tamoxifen resistance is still unclear. It’s possible that the presence of cancer stem cell confers a drugs resistance. *In vitro* studies documented that cancer stem cell exert antiapoptotic effect on breast cancer cells and counteract cell-cycle changes caused by tamoxifen, so promoting tumor growth and invasiveness (Wang et al. 2012b; Lin et al. 2013).

Aromatase inhibitors are used as second-line therapy or as first-line adjuvant therapy, but they have the disadvantage to inhibit indiscriminately aromatases, including those in bone and brain tissues, with adverse effects in terms of bone mineralization and cognitive function, respectively (Rubin et al. 2000). An ideal goal is to develop a tissue-selective aromatase inhibitor. In these sense, ASCs potentially retain many of the attributes for an optimal cellular vehicle (Rubin et al. 2000). Additional studies need to document efficacy and safety of engineered ASCs before their application in clinical trials.

## Conclusions

Current evidence sustains that ASCs represent a promising tool for innovative therapies in regenerative surgery and play a significant role in lipofilling-mediated breast reconstruction after breast cancer surgery. SVF/ASCs enrichment seems to favor long-term fat graft maintenance in reconstruction of tissue defects, likely promoting vascularization and collagen synthesis. Preliminary studies *in vivo* seem to confirm the efficacy of SVF/ASCs enrichment and the beneficial additional use of autologous platelet-derived growth factors and hormones in breast reconstruction. The improvement in long-term maintenance strongly supports the additional combined use of fat grafts with autologous platelet-derived growth factors and hormones, such as insulin. However, additional translational research studies are needed to better clarify the possible impact of these procedures on tumor microenvironment, in particular their potential effect on cancer cells. Different studies confirmed the complex and dynamic interplay between cancer cells and resident ASCs. Latters, in the tumor microenvironment, seem to affect only active cancer cells, so promoting neoangiogenesis, matrix remodeling and intercellular communication via gap-junction. In addiction, it has been hypothesized the presence of cancer stem cells, from resident stem cell or dedifferentiated tumor cells, that may favour the epithelial-mesenchymal transition, supporting tumor growth and invasiveness. In addition, the interaction between grafted ASCs and resting cancer cells doesn’t seem to be responsible for cancer recurrence because resting cancer cells are more resistant to apoptosis and they don’t require stroma or vascular structure for their survival. Preliminary data describe that SVF/ASCs enrichment did not show increased risk of new cancer or relapse compared with control group.

Finally, ASCs characteristics appear promising for their engineered use as “carrier” of adjuvant chemotherapeutic agents against residual breast cancer cells. So, the growth of malignant cells may be counteracted by local release of drugs in tumor microenvironment while systemic plasma concentration remain low, avoiding the problems related to toxicity and short life.
